# Minimally invasive surgery total knee arthroplasty is less popular, but the prosthesis designed specifically for MIS provides good survival and PROMs with a minimum follow-up of 10 years

**DOI:** 10.1186/s13018-021-02254-3

**Published:** 2021-01-29

**Authors:** Shinya Toyoda, Takao Kaneko, Yuta Mochizuki, Masaru Hada, Kazutaka Takada, Hiroyasu Ikegami, Yoshiro Musha

**Affiliations:** Department of Orthopedic Surgery, Toho University School of Medicine, 2-22-36 Ohashi, Meguro-ku, Tokyo, 153-8515 Japan

**Keywords:** Minimally invasive surgery, Total knee arthroplasty, Survival, Minimum follow-up of 10 years

## Abstract

**Background:**

The concept of minimally invasive surgery (MIS) was introduced in total knee arthroplasty (TKA) in the late 1990s. The number of MIS TKAs has clearly decreased in recent years. An implant designed specifically for MIS TKA has been used all over the world, but there are no reports of long-term postoperative results. The purpose of this study was to characterize long-term clinical results with a minimum follow-up of 10 years.

**Methods:**

This retrospective study included 109 consecutive patients with 143 NexGen CR-Flex prostheses, which are MIS tibial component prostheses designed specifically for MIS TKA. Twelve-year survival analysis was performed using Kaplan-Meier method. Revision surgery for any reason was the endpoint. Long-term clinical and radiographic results of 74 knees (55%) in 60 patients with more than 10 years of follow-up were analyzed.

**Results:**

The cumulative survival rate of the single-radius posterior-stabilized TKA of 74 knees was 94.7% (95% confidence interval, 90–99%) at 12 years after surgery. Seven knees (9%) required additional surgery during the 10-year follow-up because of periprosthetic infections. Mean postoperative Knee Society knee score and functional score were 91 and 74 points, respectively. There were no cases of prosthesis breakage, polyethylene wear, or aseptic loosening of the prosthesis.

**Conclusion:**

The prosthesis designed specifically for MIS TKA is associated with good survival and clinical results with a minimum follow-up of 10 years, even though MIS TKA has become less popular.

**Level of evidence:**

III

## Background

**Fig. 1:**
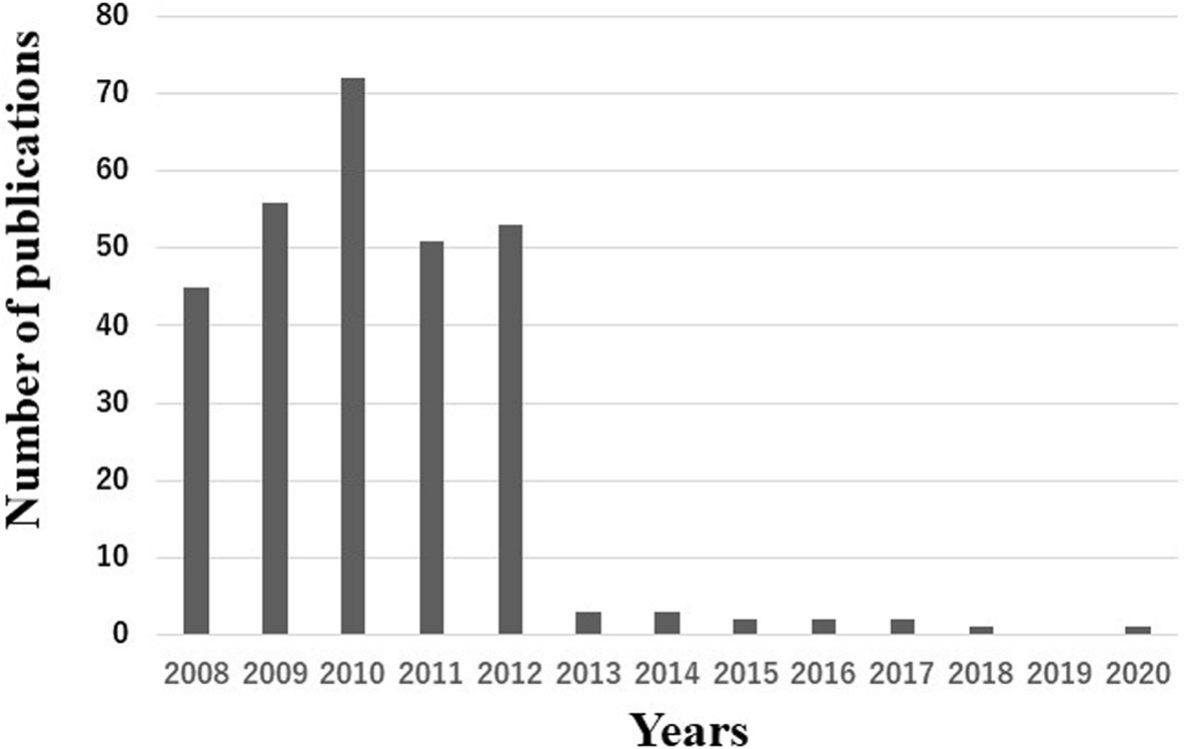
A histogram showing the number of publications on minimally invasive surgery total knee arthroplasty. There have been few publications on minimally invasive surgery total knee arthroplasty in recent years

The concept of minimally invasive surgery (MIS) was introduced in total knee arthroplasty (TKA) in the late 1990s. MIS TKA uses a smaller incision, typically does not require eversion of the patella, and involves less quadriceps splitting, which minimizes quadriceps damage [1]. It is believed to reduce hospitalization time [2, 3], increase range of motion [3, 4], reduce pain [2, 3], and restore function more rapidly [2]. However, the mid-term results reported so far have not shown a significant difference between MIS TKA and the standard approach [5–10]. The National Joint Registry (NJR) of England and Wales showed that 2.5% of TKAs were done via MIS in 2014, down from 3.8% in 2009 when MIS TKA reached its peak popularity [11]. The number of MIS TKA procedures in England and Wales recorded in the NJR clearly shows a decrease over recent years [12]. Searching for MIS TKA on PubMed shows that the number of publications has decreased sharply since 2012 (Fig. 1). An implant designed specifically for MIS has been used all over the world, but there are no reports of long-term postoperative results.

The aim of this study was to characterize long-term patient-reported outcome measurements (PROMs) and the rate of revision among patients who underwent MIS TKA and have a minimum follow-up of 10 years.

## Methods

This retrospective study included 109 consecutive patients with 143 prostheses designed specifically for MIS TKA who underwent the procedure at our institution between 2008 and 2010. There were 87 women (111 knees) and 22 men (32 knees). Mean age of patients at MIS TKA was 66.4 years (range, 58–75 years). The patients in this study were followed for a mean of 10.4 years (range, 10–12.8 years). All 143 knees were included in the survival analysis.

**Fig. 2:**
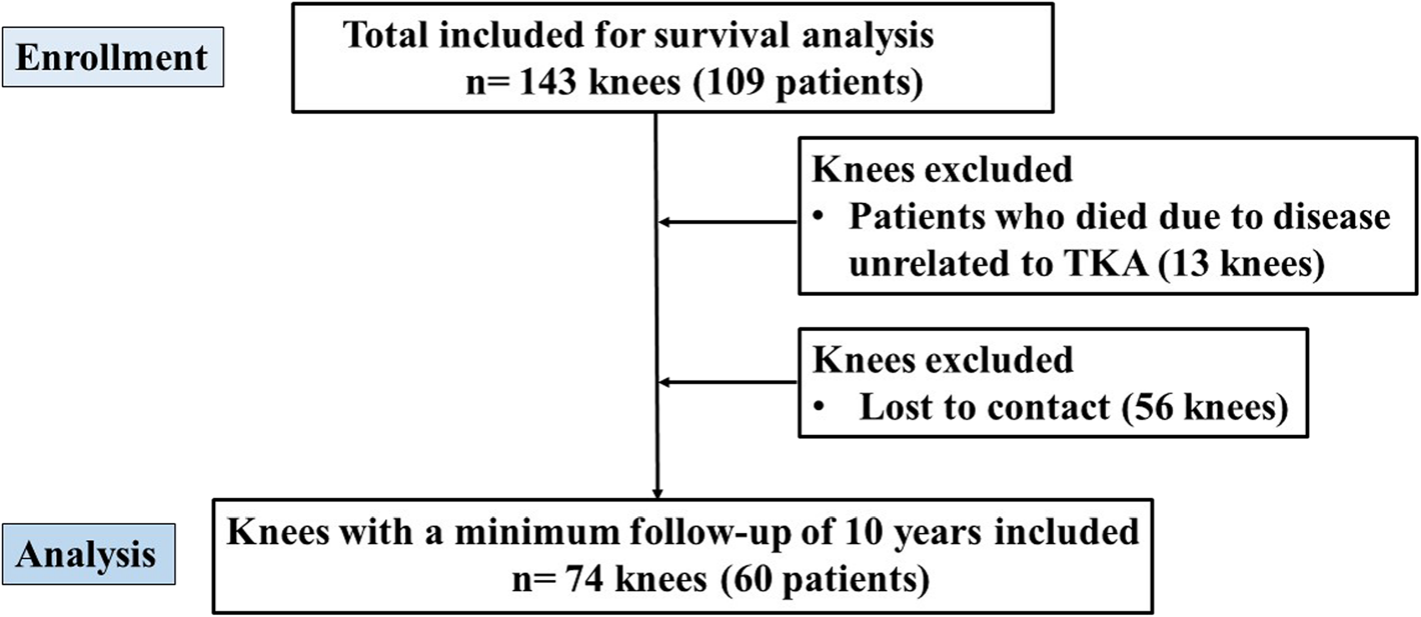
Flowchart detailing the study inclusion and exclusion criteria

To analyze clinical and radiographic outcomes at a mean of 10.4 years after MIS TKA, 69 knees were excluded because of death due to disease unrelated to TKA (13 knees) and loss to follow-up (56 knees). Consequently, 74 knees (55%) in 60 patients who had a minimum follow-up of 10 years were included (Fig. 2). There were 52 women (63 knees) and 8 men (11 knees). Mean age of patients at initial surgery was 67.4 years (range, 58–75 years). Mean follow-up was 10.3 years (range, 10–12.8 years). Mean body mass index (BMI) was 23.9 kg/m^2^ (range, 19.1–32.4 kg/m^2^).

Preoperative diagnoses included degenerative varus osteoarthritis. Exclusion criteria included valgus knee deformities, severe fixed flexion contractures of more than 15° of flexion, severe extra-articular deformities, prior high tibial osteotomy, and knee joint infection.

MIS TKA was performed using the measured resection technique and cemented technique by one author. After inflating the tourniquet to 300 mmHg at the beginning of the procedure, subvastus arthrotomy was performed. Distal femoral osteotomy was performed at a valgus angle based on preoperative radiographs using an intramedullary resection guide. Rotational alignment was adjusted to the surgical epi-condylar axis of the femur. The anterior referencing technique was used for the posterior femoral cut. The size of the femoral component was determined based on the anteroposterior length of the femur, which was independent of the flexion gap. An extramedullary resection guide was used for proximal tibial osteotomy. The angle of the osteotomy was intended to be perpendicular to the mechanical axis and to recreate preoperative posterior inclination of the tibia based on measurements from preoperative radiographs in the coronal and sagittal planes.

Rotational alignment of the tibia was defined as the anteroposterior axis between the footprint of the posterior cruciate ligament and the medial border of the patellar tendon [13]. The patella was resurfaced during all MIS TKA procedures. For medial ligament balancing in extension, the deep layer of the medial collateral ligament (MCL) was released within 1 cm from the joint line for bone resection and surrounding osteophytes were removed. The superficial layer of the MCL, semimembranosus, and posterior oblique ligament were not released. On the first day after MIS TKA, weight-bearing was not restricted and patients were allowed to walk with or without assistive devices after the drainage tube was removed.

**Fig. 3:**
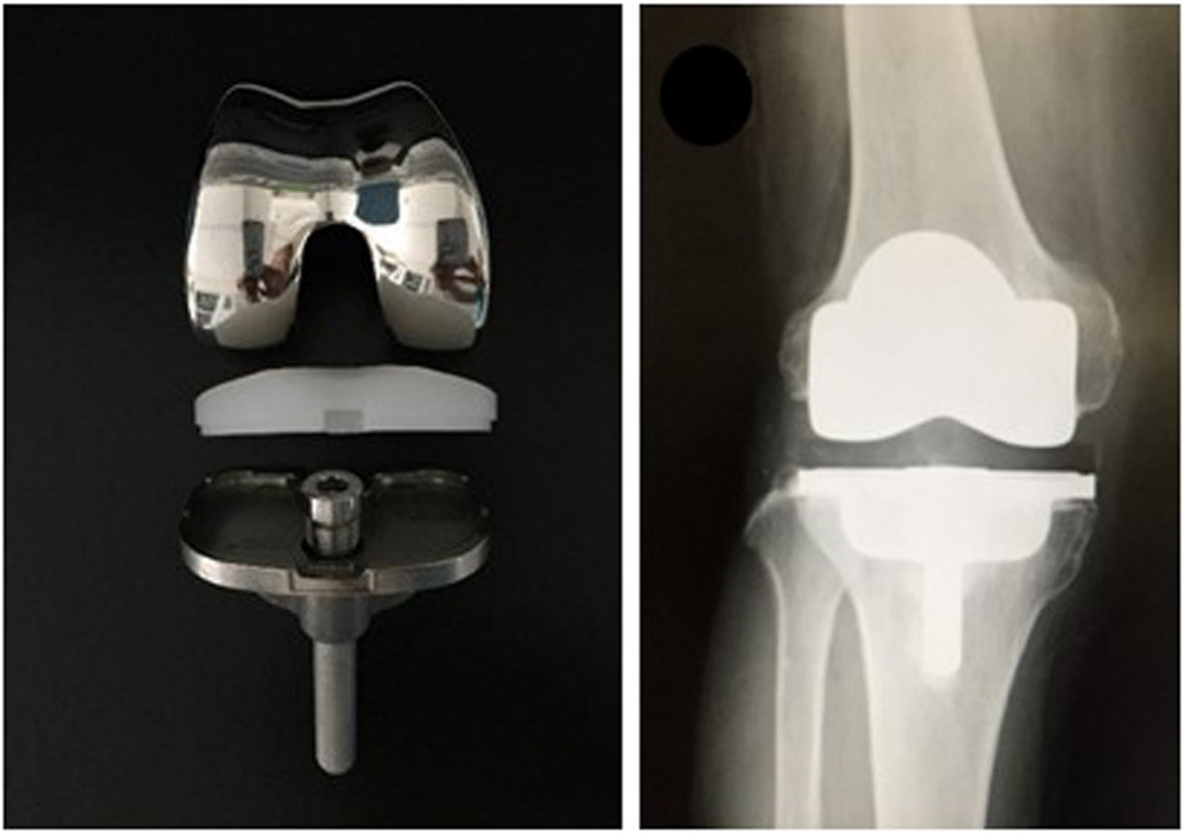
Photograph of a NexGen CR-Flex femoral component, fixed bearing, MIS modular tibial component with a small keel, and drop down stem extension (Zimmer Biomet, Warsaw, IN, USA)

A NexGen cruciate retaining (CR)-Flex femoral component and fixed bearing (NexGen CR-Flex, Femoral Component; Zimmer Biomet, Warsaw, IN, USA) were used. On the tibial side, a NexGen MIS modular tibial component with a 45-mm or 75-mm drop down stem extension (NexGen CR-Flex, MIS tibial Component; Zimmer Biomet, Warsaw, IN, USA) was used in all MIS TKA procedures (Fig. 3). The characteristics of the prosthesis allowed for implant insertion without tibiofemoral dislocation and placement of a stem extension after positioning the tibial component in extension.

**Fig. 4:**
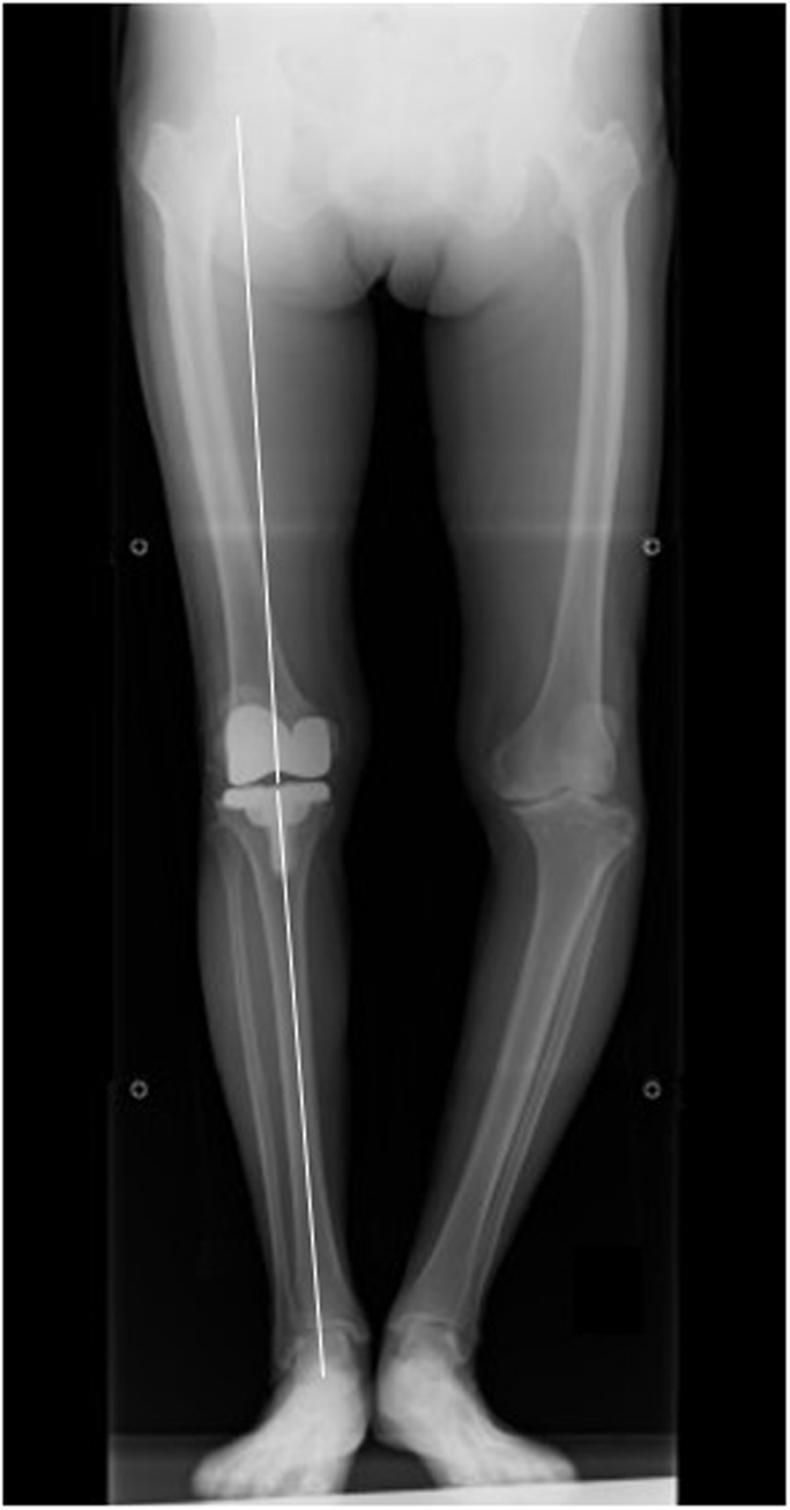
Postoperative anteroposterior radiograph of the lower extremity in the standing position. Hip-knee-ankle angle is 0°

Patient demographics, including gender, age, height, weight, BMI, and length of follow-up were recorded. Range of motion (ROM) was passively measured with a long-arm goniometer in the supine position. Preoperative and postoperative anteroposterior radiographs of the lower extremity in the standing position were taken using a long film (Fig. 4). All postoperative radiographs were taken within 2 weeks of the operation. Assessments of component positioning were performed in accordance with the roentgenographic knee evaluation system endorsed by the Knee Society [14]. The following angles were evaluated: the medial angle between the distal surface of the femoral component and the coronal anatomical axis of the femoral shaft (*α*), the medial angle between the tibial plate and the coronal mechanical axis of the tibial shaft (*β*), the flexion angle between the sagittal axis of the femoral component and the sagittal anatomical axis of the femoral shaft (*γ*), and the posterior titling angle between the tibial plate and the sagittal mechanical axis of the tibial shaft (*δ*).

The presence and location of radiolucent lines at the bone-cement interface were assessed according to Knee Society guideline [14] at last postoperatively follow-up. At the same time, radiolucent lines in each zone were evaluated according to the method of Ranawat et al. [15]. Survival analysis was performed to determine the cumulative survival rate of the prosthesis. The endpoint for the analysis was revision for any reason.

Knee Society Knee and Function scores (1989 KSKS and KSFS, respectively) based on the 1989 Knee Society clinical rating system [14] were measured before surgery. In addition to 1989 KSKS and KSFS for comparison between pre- and post-operation, four patient-reported sections (symptoms, patient satisfaction, patient expectations, standard and advanced activities) from the 2011 Knee Society Score (2011 KSS) [16], three patient-reported sections (pain, stiffness, and physical function) from the Western Ontario and McMaster Universities osteoarthritis Index (WOMAC) [17], 12 questionnaire of Forgotten Joint Score (FJS-12) [18], and four patient-reported sections (anterior knee pain, quadriceps strength, ability to rise from chair, and stair-climbing) from the Patella score [19] were measured at last postoperative follow-up.

The study protocol was approved by the Ethics Committee of Toho University Ohashi Medical Center (H20074). All patients provided informed consent for participation.

Means and standard deviations were used to describe the data. A paired *t* test was performed in order to compare preoperative and postoperative ROM. Kaplan-Meier survival analysis was used to determine the cumulative rate of prosthesis survival during the study period. SPSS version 24.0 (SPSS Inc., Chicago, IL, USA) was used for statistical analyses. *p* < 0.01 was considered statistically significant.

## Results

**Fig. 5:**
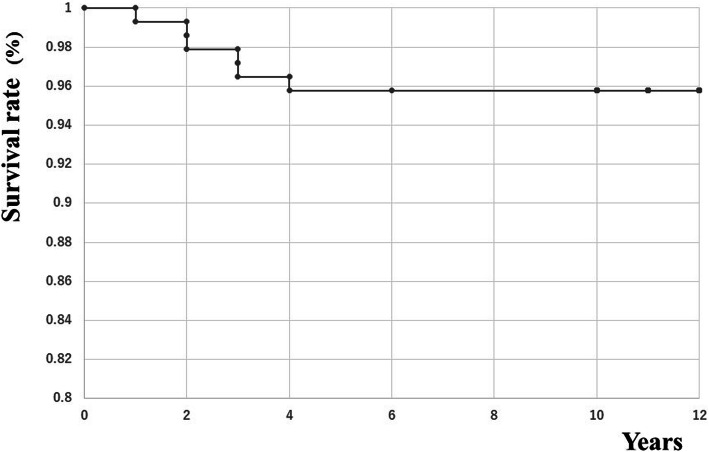
In the Kaplan-Meier analysis, the projected rate of survival to revision as the endpoint was 94.7% at 12 years (95% confidence interval 0.90–0.99)

The cumulative rate of survival of revision at end point was 94.7% at 12 years after MIS TKA (95% confidence interval, 0.90–0.99; Fig. 5).

Seventy-four knees (60 patients) had an average 1989 KSKS of 91 points and 1989 KSFS of 74 points at the last follow-up. At the last follow-up examination, 1989 KSKS and KSFS were significantly higher (*p* < 0.01) (Table 1). Mean extension and flexion angle were significantly improved postoperatively (Table 1). Seven knees (9%) had prosthetic joint infection (Leone classification [20]; type 2, one knee; type 3, four knees; type 4, two knees). These patients underwent a two-stage revision procedure.

**Table 1:** Preoperative and postoperative Knee Society score and range of motion

Variable	Preoperative	Final follow-up	*p* value
1989 Knee society knee score (points)*	10 ± 12	91 ± 9	< 0.01
1989 Knee society function score (points)*	38 ± 11	74 ± 20	< 0.01
Range of motion*			
Extension angle (°)	9 ± 7	1 ± 2	< 0.01
Flexion angle (°)	106 ± 18	124 ± 11	< 0.01

*Data are presented as means ± standard deviation

**Fig. 6:**
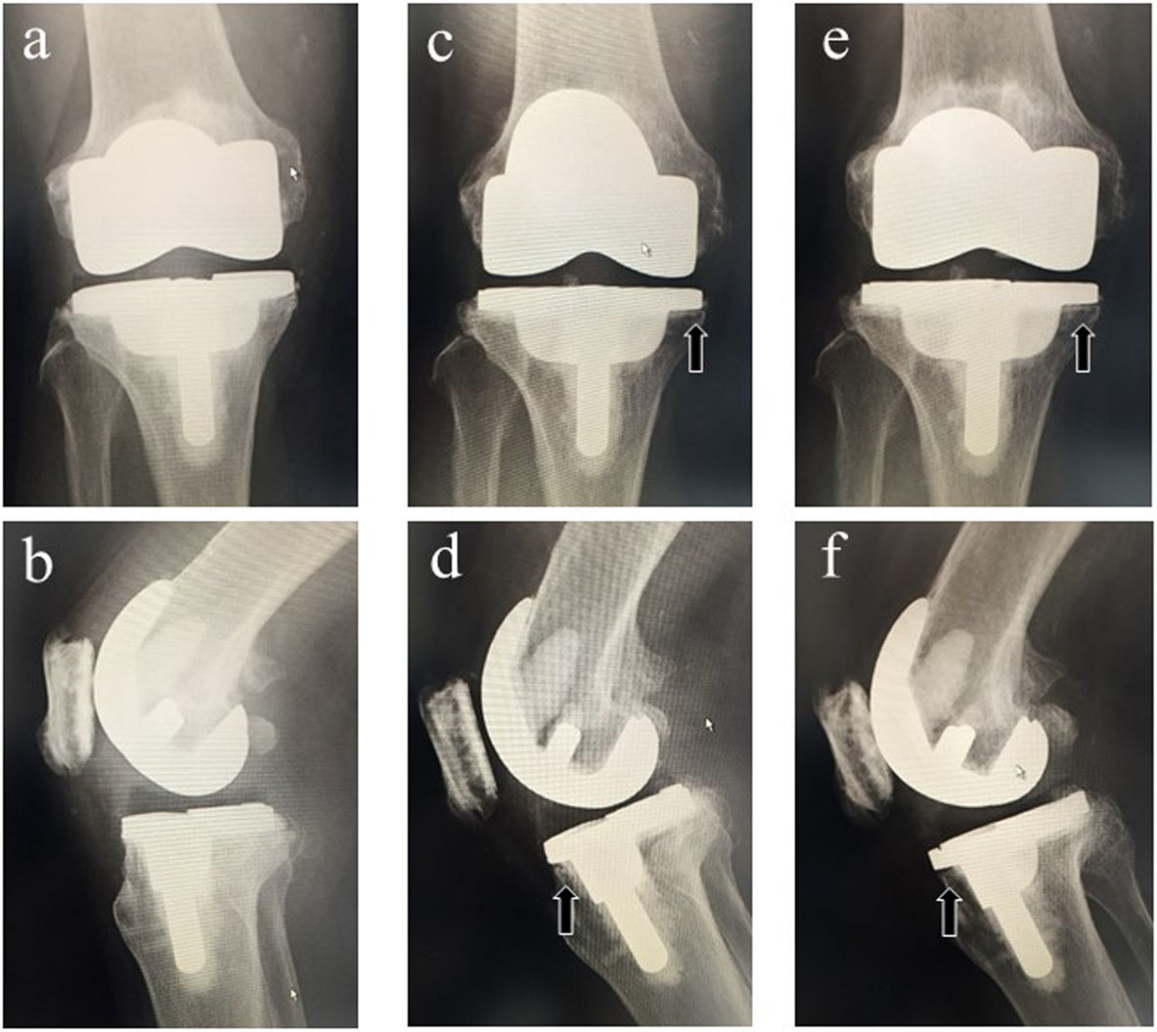
Coronal and sagittal views of radiographs in a same patient at 2 weeks (**a**, **b**), 6 years (**c**, **d**), and 11 years (**e**, **f**) postoperatively. Radiolucent lines (black arrows) were identified 6 years after surgery, but these were not progressive in nature and migration of the components was not observed

All patients underwent complete radiological follow-up examinations. Prosthetic alignment is shown in Table 2. Radiolucent lines < 1 mm were identified around the femoral component in 11 knees (15.0%) and around the tibial component in 21 knees (28.0%). Radiolucent lines were in zone 1 for 14 knees and zone 4 for 5 knees in the tibia on the anteroposterior projection; zone 2 for 6 knees in the tibia on the lateral projection; and zone 1 for 8 knees and zone 4 for 2 knees in the femur on the lateral projection. These radiolucent lines were not progressive in nature and migration of the implant components was not observed (Fig. 6). There were no cases of prosthesis breakage, polyethylene (PE) wear, or aseptic loosening of the prosthesis. Based on PROMs at the last postoperative follow-up, 46 knees (62.1%) had a patient satisfaction score of more than 80% (Table 3).

**Table 2:** Preoperative and postoperative radiographic results

Variable	Value
Tibiofemoral angle (°)*	
Preoperative	11.1 ± 7.9 (varus)
Postoperative	4.3 ± 1.7 (valgus)
Postoperative prosthetic alignment (°)*	
*α*	95.9 ± 2.3
*β*	89.2 ± 2.1
*γ*	1.9 ± 2.5
*δ*	82.1 ± 7.3

*Data are presented as means ± standard deviation

**Table 3:** Postoperative patient-reported outcome measurement

Variable	Value
2011 Knee Society Score (points)*	
Symptoms (25)	19.0 ± 4.5
Patient satisfaction (40)	25.3 ± 8.5
Patient expectations (15)	10.4 ± 3.6
Standard and advanced activities (100)	64.9 ± 21.7
WOMAC (points)*	
Pain (20)	13.3 ± 5.9
Stiffness (8)	5.1 ± 2.4
Physical function (68)	38.7 ± 16.0
FJS-12 (points) (100)*	66.5 ± 20.2
Patella score (points)*	
Anterior knee pain (15)	11.7 ± 4.2
Quadriceps strength (5)	3.1 ± 1.8
Ability to rise from chair (5)	3.7 ± 1.4
Stair-climbing (5)	3.8 ± 1.5

*Data are presented as means ± standard deviation

*FJS-12* Forgotten Joint Score-12 questionnaire, *WOMAC* Western Ontario and McMaster Universities Osteoarthritis Index

## Discussion

The most important findings of this study were that the prosthesis designed specifically for MIS TKA provided had good survival, with no cases of prosthesis-related revision and good PROMs at a minimum follow-up of 10 years, despite the fact that the MIS TKA has not received much attention in recent years.

To the best of our knowledge, this is the first study to evaluate the survival rate of the prosthesis designed specifically for MIS TKA and long-term PROMs with a minimum follow-up of 10 years.

MIS TKA took the world by storm after Tria et al. described the quadriceps sparing (QS) method [21]. It was heralded as a completely non-invasive invasion of vastus medialis oblique (VMO). However, this technique was theoretically unviable in the majority of type 3 patients with VMO adhering to the median patella [1, 22, 23]. Since these reports, the significance of the QS method has been questioned. On the other hand, the goals of MIS-TKA are now clear. MIS-TKA is not just an operation with a small incision, but a quadriceps-friendly operation that aims to preserve the knee extension mechanism, which is consistent with the non-eversion patella and mobile window technique. Leopold [1] reported the definition of MIS TKA, which involves six features: (1) minimal separation of the knee extension mechanism, (2) no tipping of the patella, (3) no dislocation of the femorotibial joint, (4) osteotomy with a small guide for MIS, (5) mobile window technique, and (6) a small skin incision of approximately 10 cm. However, a systematic review found that the advantages of MIS TKA may be offset by longer operative times and poor implant placement [24]. Randomized control trials did not find that MIS TKA had any advantages over conventional TKA except for smaller skin incision [25, 26]. The evidence that has accumulated from 2003 to 2012 suggests that the benefits of MIS TKA within 3 months of surgery are early functional recovery and smaller skin resection. Therefore, the number of MIS procedures is currently decreasing. We believe that patients who will benefit from a minimally invasive procedure have not changed in the past and will not change in the present.

A recent report of TKA with a minimum follow-up of 10 years is shown in Table 4 [27–35]. The clinical outcomes described in these reports were comparable to the results of the present study. Cumulative survival was the third worst after Genesis I, the implant used in the report by Victor et al. [35], and Trekking PS, the implant used in the report by Serna et al. [27] The reason for the low cumulative survival rate in this study is that all patients who underwent revision surgery had diabetes (HbA1c > 6.6%) and poor perioperative glycemic control. No patients underwent revision for prosthesis breakage, polyethylene (PE) wear, or aseptic loosening of the prosthesis.

**Table 4:** Mean age, mean follow-up, and clinical outcomes using various prostheses

Prosthesis	Mean age	Mean follow-up (years)	Survival rate (%)	Mean ROM (°)	Mean KSKS (points)	Mean KSFS (points)	Reference
**Trekking CR (fixed)**	**68.8**	**10**	**95.7**	**101.2**	**86.4**	**84.4**	**Serna et al. [28]**
**Trekking PS (fixed)**	**70.1**	**10**	**92.7**	**100.7**	**85.2**	**85.6**	**Serna et al. [28]**
**Duracon (fixed)**	**63**	**11**	**95.6**	**115**	**84**	**73**	**Jauregui et al. [29]**
**NexGen LPS-Flex (mobile)**	**61.5**	**11.2**	**99.5**	**131**	**92**	**80**	**Kim et al. [30]**
**PFC Sigma PS (mobile)**	**73**	**11.5**	**96.6**	**106**	**70.1**	**58**	**Ulivi et al. [31]**
**Scorpio PS (fixed)**	**68**	**8**	**97.7**	**129**	**97**	**75**	**Chang et al. [32]**
**AGC PS (fixed)**	**69.6**	**8.7**	**98.7**	**115**	**85**	**77**	**Faris et al. [33]**
**Genesis I (CR or PS fixed)**	**69.3**	**11**	**90.1**	-	**KSS 81**	-	**Victor et al. [34]**
**Genesis II (CR or PS fixed)**	**66**	**11**	**98.1**	-	**KSS 83**	-	**Victor et al. [34]**
**MPK. Alumina (fixed)**	**72**	**10**	**99.1**	**116**	**89**	**68**	**Nakamura et al. [35]**
**NexGen LPS (fixed)**	**65.3**	**15.6**	**98.7**	**128**	**KSS 93**	-	**Kim et al. [36]**
**NexGen LPS-Flex (fixed)**	**65.3**	**15.6**	**98.4**	**127**	**KSS 92**	-	**Kim et al. [36]**
**MIS NexGen CR-Flex (fixed)**	**67.4**	**10.3**	**94.7**	**124**	**91**	**74**	**The current study**

*ROM* range of motion, *KSKS* knee society knee score, *KSFS* knee society function score, *KSS* knee society score

Victor et al. [35] found a significant difference in survival between thinner and thicker PE inserts. Insert size > 11 mm had low survival (56.7%) at 14 years. In this study, PE thickness was not affected. Bonutti et al. [36] first reported favorable clinical and radiological results using a modular tibial implant (NexGen LPS-Flex). The 9-year survival rate was 97.1% in 90 knees. Benazzo and Rossi [37] reported on the results of MIS TKA using NexGen CR-Flex, MIS tibial Component. The 5-year survival rate in that prospective study was 97.9% in 200 knees during mean 3 years of follow-up. Yoo et al. [38] found that the survival rate was 99.4% with a minimum follow-up of 5 years and there were no prosthesis-related revisions. Yang et al. [39] evaluated the efficacy and longevity of a modular tibial implant in MIS TKA and compared the difference between CR and posterior-stabilized (PS) designs. They reported that the survival rate was 97.8% and 95% with CR and PS designs, respectively, at 5 years of follow-up and that MIS TKA with both CR and PS prostheses achieved similarly good clinical results.

The present study had the longest follow-up period, with a mean follow-up of 10.4 years, among studies of the prosthesis designed specifically for MIS TKA. The survival rate was 94.7% at 12 years and there were no cases of prosthesis-related revision.

The current study has several limitations. First, there was no control group. Therefore, it is not clear whether PROMs at a minimum follow-up of 10 years were good or not. The 2011 KSS and FJS-12 were proposed less than 10 years ago. Second, the study design was retrospective and the sample size was relatively small. Therefore, we cannot discuss whether MIS TKA and the prostheses designed for MIS is better than others. To mitigate this limitation, we evaluated long-term prosthesis survival in consecutive patients. The results of the present study were comparable to recent reports of conventional TKA with a minimum follow-up of 10 years (Table 4). Third, the vast majority of patients were women. Thus, caution is needed while comparing our findings to those of other studies with different sex distribution. However, female pre-dominance among patients undergoing TKA is common in Asian countries [40].

## Conclusion

In conclusion, despite the decreasing popularity of MIS TKA with NexGen CR-Flex, this tibial component designed specifically for MIS TKA had satisfactory long-term clinical and radiographic outcomes and good survival.

## Data Availability

All data generated or analyzed during this study are included in this published article.

## Abbreviations

BMI: Body mass index
CR: Cruciate retaining
FJS: Forgotten joint score
KSFS: Knee Society function score
KSKS: Knee Society knee score
KSS: Knee Society score
MCL: Medial collateral ligament
MIS: Minimally invasive surgery
NJR: National joint registry
PE: Polyethylene
PROM: Patient-reported outcome measurement
PS: Posterior-stabilized
QS: Quadriceps sparing
ROM: Range of motion
TKA: Total knee arthroplasty
VMO: Vastus medialis oblique
WOMAC: Western Ontario and McMaster Universities osteoarthritis Index
